# Neutralizing Antibody Response after Intramuscular Purified Vero Cell Rabies Vaccination (PVRV) in Iranian Patients with Specific Medical Conditions

**DOI:** 10.1371/journal.pone.0139171

**Published:** 2015-10-06

**Authors:** Pooneh Rahimi, RouhAllah Vahabpour, Mohammad Reza Aghasadeghi, Syed Mehdi Sadat, Nader Howaizi, Ehsan Mostafavi, Ali Eslamifar, Vida Fallahian

**Affiliations:** 1 Department of Hepatitis and AIDS, Pasteur Institute, Tehran, Iran; 2 WHO Collaborating Center for Reference and Research on Rabies, Pasteur Institute, Tehran, Iran; 3 Department of Vaccination, Rabies post-exposure prophylaxis, Pasteur Institute, Tehran, Iran; 4 Department of Epidemiology, Pasteur Institute, Tehran, Iran; Indian Institute of Science, INDIA

## Abstract

**Objective:**

Post exposure prophylaxis using one of the WHO-approved vaccines is the method of choice for preventing rabies. Abnormal immune function in patients with some specific medical conditions, such as pregnancy, chronic hepatitis B virus infection, different types of cancers like lymphoma, diabetes I and II, corticosteroid consumption by patients with rheumatoid arthritis and lupus erythematosus, could impair the immunologic response to various vaccines. The immune response to rabies vaccination has never been examined in patients with any of these described medical conditions. This study purposed to evaluate the neutralyzing antibody response after vaccination with purified Vero cell rabies vaccine (PVRV) according to the WHO-recommended Post–Exposure Prophylaxis (PEP) "ESSEN" regimen.

**Methods:**

Thirty healthy volunteers and 50 volunteers with different medical conditions who were exposed to a suspected rabid animal in the 2nd or 3rd category of exposure received 5 doses of PVRV under the ESSEN protocol. Three blood samples were collected on days 0 (before the first dose), 14, and 35. The anti-rabies antibody titer was measured using the Rapid Fluorescent Foci Inhibition Test (RFFIT) and an ELISA Bio-Rad, Platelia, Rabies II kit.

**Results:**

All subjects reached NAb titers above 0.5 IU/ml by day 14 after vaccination. On day 35 (1 week after receiving the last rabies vaccine), anti-rabies antibodies were in the protective level (>0.5 IU/ml) in both groups. There was no statistically significant difference in anti-rabies antibody response due to the type of exposure (category 2 or 3), and successful seroconversion was confirmed in both groups.

**Conclusion:**

In conclusion, the ESSEN protocol using the PVRV vaccine is sufficient for rabies prophylaxis in patients with specific medical conditions.

## Introduction

Rabies is a viral encephalitis caused by RNA viruses in the Family *Rhabdoviridae*, Genus *Lyssavirus*. It has a high mortality rate and is usually transmitted by a bite or scratch from a rabid animal to humans or other animals [1]. Although rabies is a preventable fatal disease, it remains a serious public health problem in many developing countries. At least 60,000 human deaths and 10 million post-exposure treatments are reported each year throughout the world [2].

In rabies-endemic countries like Iran, an animal is presumed rabid; therefore, each exposure to an animal leads to post-exposure vaccination therapy [3]. Post-exposure treatment depends on the type of exposure and consists of no treatment for category I, vaccine alone for category II, and immediate treatment by vaccination therapy with rabies specific immunoglobulin for category III [2, 4]. One of the most recommended post-exposure prophylaxis protocols (ESSEN protocol) includes five single doses of vaccine over a 28-day period with intramuscular (IM) administration of cell culture rabies vaccines recommended by WHO [5, 6]. Inducing a quick response as soon as possible after exposure to the rabies virus to prevent its progress towards the central nervous system is the most critical criterion for the effectiveness of any post-exposure therapy. Although use of the ESSEN regimen has reduced considerably the number of human deaths due to rabies in Iran, still, some poor patient compliance with the vaccination schedule exists and results in death. Recommendations for PEP in unvaccinated persons depend on the immune status. The current 5-dose regimen should still be recommended in immunosuppressed persons [7, 8]. Immunosuppression might be identified as a variety of conditions, such as congenital immunodeficiency, HIV infection, AIDS, bone marrow transplant, malignancies and cancers (leukemia, lymphoma), and certain other medical conditions, such as renal failure, diabetes, or cirrhosis. Therapy with corticosteroids, antimetabolites, radiation, and alkylating agents also cause patients to be immunocompromised which may dampen the immune response to vaccines [9–12]. Herein, the authors tried to accommodate the collection of information on the effectiveness of PEP under the ESSEN protocol using PVRV in some immunocompromising conditions.

## Materials and Methods

### Patients

Participants in the study (from 2012 to 2014) included 30 healthy volunteers and 50 patients with different types of specific medical conditions, such as pregnancy, diabetes I or II, chronic infection with the hepatitis B virus, different types of cancer such as lymphoma, and those who were immunocompromised due to receiving corticosteroids such as rheumatoid arthritis patients and lupus erythematosus patients. All participants had been exposed to rabies belonging to the WHO categories II or III through animal bites (mostly dog). In all cases, the biting animal was not traceable, so its rabies status could not be confirmed. Per the Helsinki Declaration, the aim of the project and the blood sampling procedures were explained clearly to each participant. Then, the questionnaire and the informed consent form were signed by each volunteer or volunteer’s custodian. This study was approved by the Ethics Committee of Pasteur Institute of Iran.

No participant had a history of rabies vaccination in the prior 20 years. Patients with special medical conditions had had the specific condition for at least one year before exposure. All participants received the standard PEP, including wound treatment and injection of 0.5 ml intramuscular anti-rabies vaccine (PVRV: purified vero cell vaccine, manufactured by Sanofi Pasteur, France, with potency of 5 IU per ampule) under the ESSEN protocol in the Prevention and Treatment of Rabies Center, Department of Vaccination, Pasteur Institute of Iran. Human rabies immune globulin (HRIG) (20 IU/kg body weight) was also given to all patients on day 0.

Inclusion criteria were aged 18–65 years, willing to give blood samples on stipulated dates, and being available for follow-up for at least 2 months post-vaccination. Exclusion criteria included having a history of rabies vaccination or any animal bite, recent immunization with either killed or live vaccines within 3 weeks prior to the rabies vaccination, receiving anti-malarial drugs, and having acute febrile disease.

### Immune response assessment

Blood samples for determining rabies neutralizing antibodies (NAb) were obtained before the first dose of the rabies vaccination (day 0) and on days 14 and 35 (1 week after the last vaccination). The rabies neutralizing antibody titer was measured using the Rapid Fluorescent Focus Inhibition Test (RFFIT) as described by WHO [13, 14]. The results of antibody titers were expressed in IU/ml in comparison with the international reference anti-rabies immunoglobulin serum with a known potency of 30 IU that had been diluted to a potency of 2.0 IU/ml. A positive serum control standard diluted to a potency of 0.5 IU/ml and a negative serum control standard with a potency of <0.1 IU/ml were prepared and included in each test. Briefly, BSR cells, a clone of Baby Hamster Kidney Cell line (BHK-21), were grown in Eagle’s minimum essential medium supplemented with 10% fetal bovine serum (EMEM-10) and used in tests as previously described [13]. Serum samples were diluted at 1/3 in 96-well microplates and mixed with a constant dose of challenge virus that caused infection in 80% of the cells, as described [13].

The sera-virus mixture was incubated at 37°C for 1 h. After incubation, susceptible cells were added to the serum-virus mixtures; then, they were incubated for 24 h. After that, the cell monolayer was acetone fixed and stained using the anti-rabies nucleocapsid conjugate (Bio-Rad, USA) according to the manufacturer`s instructions to detect the presence of non-neutralized rabies virus (fluorescent foci). Calculations were made using the Reed and Muench method [13]. A titer of ≥0.5 IU/ml antibody was considered "protective" against rabies. Anti-rabies neutralizing antibody titer (EU)/ml) was also determined by ELISA (Bio-Rad, Platelia, Rabies II, USA) according to the manufacturer`s instructions. ELISA is based on the extracted rabies virus glycoprotein and a peroxidase conjugate (protein A from staphylococcus aureus).

### Statistical Analysis

Data from both laboratory methods used in measuring anti-rabies antibody titer was analyzed using the repeated measure analysis of variance. Sex and age were considered as the covariates in this study; p-values <0.05 were considered significant.

## Results

Thirty healthy volunteers and 50 patients with specific medical conditions were recruited for the study; their medical conditions have been described in Table 1.

**Table 1:** Frequency of volunteer participants (healthy volunteers and patients group) in this study with descriptions of special medical conditions in patients volunteers.

Health conditions of volunteer participants	Diseases	Valid Percent		Cumulative Percent
Normal	Normal	30	100.0	100.0
Patient	Cancer	5	10	10
	Diabetes1	6	12	22
	Diabetes2	20	40	62
	Hepatitis B	2	4	66
	Lymphoma	2	4	70
	Lupus	4	8	78
	Pregnant	6	12	82
	Rheumatoid arthritis	5	10	92
	Total	50	100.0	100.0

Males made up 62.5% and females 37.5% of the patients group, and healthy volunteers were 83.3% males and 16.7% females. Patients and healthy volunteers had mean ages of 41.7 and 29.3 years, respectively.

No participant had any detectable anti-rabies antibody titer prior to vaccination. No serious adverse reactions were observed in this study. For example, no pregnant women reported miscarriage, stillbirth, or fetal malformation. Based on the WHO category of exposure to the rabies virus, 8 and 9 participants among patients and healthy volunteers, respectively, had exposure through animals to the rabies virus.Table 2 depicts the frequency of each type of exposure in accordance with specific diseases in patients with specific medical conditions

**Table 2:** Frequency of each type of exposure in healthy volunteers and in patients group according to their specific disease.

Health Condition of Participants	Number of Exposure Type2	Number of ExposureType 3	Total
Normal	21	9	30
Cancer	4	1	5
Diabetes1	4	2	6
Diabetes2	16	4	20
Hepatitis B	2	0	2
Lymphoma	2	0	2
Lupus	3	1	4
Pregnant	6	0	6
Rheumatoid arthritis	5	0	5
**Total**	42	8	50

There was no statistically significant difference in anti-rabies antibody response due to type of exposure (category 2 or 3) in either study group. All subjects reached NAb titers above 0.5 IU/ml by day 14 after the 2nd vaccination dose (4.2–28.1 IU/ml in healthy volunteers and 1–12.4 IU/ml in patients). Geometric MeanTiter (GMT) was measured because of the results of the RFFIT method, and the results were 16.2 IU/ml and 8.73 IU/ml in healthy volunteers and patients, respectively. Based on the results of ELISA, GMT was also calculated to be 2.16 EU/ml in healthy volunteers (anti-rabies antibody titer: 0.78–3.98 EU/ml) and 1.40 EU/ml in patients (anti-rabies antibody titer from 0.04 to 2.75 EU/ml). On day 35 (1 week after receiving the last rabies vaccine) anti-rabies antibody levels were above the protective level (>0.5 IU/ml) in both groups as measured by the RFFIT method (8.3–45.5.IU/ml in healthy volunteers and 8–30.2 IU/ml in patients) with geometric mean titers of 30.3 IU/ml and 20.7 IU/ml in healthy volunteers and patients, respectively. Using the ELISA kit, the anti-rabies antibody titer was measured as 2.75- >4 EU/ml and 1.2- >4 EU/ml in healthy volunteers and patients, respectively, and GMT was calculated as 3.77 EU/ml in healthy volunteers and 2.36 EU/ml in patients (BioRad, Platelia, Rabies II, USA). Table 3 shows the minimum and maximum anti-rabies antibody titer values measured by RFFIT and ELISA methods in patients according to their specific medical conditions. Successful seroconversion was confirmed in both groups after participants received the 3rd dose of PVRV, and it remained at the protective level 1 week after the last vaccination.

**Table 3:** Results of minimum and maximum anti-rabies antibody titer measured by RFFIT and ELISA methods in patients group according to their specific medical conditions.

* Anti-rabies Ab titer	Day14		Day14		Day35		Day 35	
	RFFIT(IU/ml)		ELISA(EU/ml)		RFFIT(IU/ml)		ELSA(EU/ml)	
**Patients	Min	Max	Min	Max	Min	Max	Min	Max
Cancer	1	11.3	0.36	2.1	8.9	30.2	1.24	>4
Diabetes 1	9.6	11	0.78	1.86	18.3	28.1	1.2	>4
Diabetes 2	1.2	12.4	0.4	2.6	8	24.3	1.4	>4
Hepatitis B	10.2	11	1.1	2.45	21.6	28.4	-	>4
Pregnant	6.4	12	0.55	2.75	18.6	28.3	1.2	>4
Rheumatoid arthritis	4.8	9.6	0.5	2.35	19	28.2	1.8	>4
Lupus	6.2	10.6	0.64	1.81	16.8	24	1.6	>4

*Anti–rabies antibody titer measured by RFFIT and ELISA methods.

** Patients group according to their specific medical conditions.

## Discussion

Vaccination is the only method for preventing rabies, which has an almost 100% fatality rate. Post-exposure vaccination is recommended for anyone exposed to rabies, especially those experiencing animal bites [1–6]. Purified rabies vaccine cultured on Vero cells (Verorab, Sanofi Pasteur) is WHO-approved for pre- and post-exposure prophylaxis by intradermal and intramuscular routes [4]. A standard neutralizing antibody titer of 0.5 IU/ml is considered an adequate immune response to the rabies vaccine [2, 15].

In this study, the difference in mean age between the 2 studied groups was statistically significant (p<0.01), possibly because participants in the patient group avoided exposure to animals because of their special medical conditions. Anti-rabies antibodies were undetectable on Day 0 in both groups, in accordance with questionnaire responses submitted by volunteers in both groups.

Several published studies on rabies PEP safety in pregnancy have all found the vaccine to be safe; therefore, pregnant women who have been bitten by a wandering animal must undergo PEP treatment [4, 16, 17]. Results of the current study are consistent with those of previous studies as the WHO protective anti-rabies antibody titer (= >0.5 IU/ml) was detected in pregnant patients with no adverse effects due to rabies vaccinations [16–20].

The effectiveness of rabies vaccination has been established for many years in healthy subjects, but responsiveness to the rabies vaccine in patients with certain medical conditions should be examined [9]. In this study the effectiveness of PEP under the ESSEN regimen with rabies vaccine (PVRV) was also assessed in chronic hepatitis B patients. Results indicated an acceptable immune response and anti-rabies antibody titer of >0.5 IU/ml in patients with chronic HBV infection as well as diabetes I and II patients. These results could not be compared with other investigations because of the lack of similar published data.

Previous studies have shown that patients with immunosuppressive conditions, such as malignancies and cancers, renal failure, diabetes, cirrhosis or HIV infection, have a poor or even undetectable NAb response to conventional pre- or post-exposure rabies vaccination [10–12]. Here, on day 14, the minimum anti-rabies antibody titer was measured in cancerous patients, and on day 35 the minimum titer was detected in patients with diabetes II. However, both measured minimum anti-rabies antibody titers indicated a protective immune response (= >0.5 IU/ml) as depicted in Table 3. Patients with cancer are considered immunocompromised, especially while receiving anti-cancer treatment such as chemotherapy or radiation therapy. During this study, some patients received anti-cancer therapy, and that might be the main reason for a weak immune response to the rabies vaccination. Although anti-rabies antibodies at the protective level were detected in patients with diabetes II on Day 35, a decline in antibody titer was also observed in these patients. It has been shown that the immune response of these patients is not as good as that of immunocompetent persons [9, 21, 22]. Because of a lack of similar published data, the results of this study could not be compared with other investigations; therefore, more studies are needed to better understand the interaction between the immune response in patients with these medical conditions and rabies vaccination.

In this study, a total of 16 immunocompromised patients (except pregnant women) had an anti- rabies NAb in their serum that reached the protective level (= >0.5 IU/ml) on Day 14 and was detectable 1 week after vaccination with PVRV under the ESSEN protocol. Many studies have shown that the ESSEN protocol with 5 doses of WHO-approved vaccines, such as PVRV, is still the best way to prevent fatalities from rabies [7, 8]. In this paper, the effectiveness and safety of PVRV under the ESSEN protocol in patients with specific medical conditions and healthy volunteers with WHO category 2nd and 3rd exposure are compared and described. For some patients, this should be considered as the first report in this field [4]. One review of experiences with PVRV worldwide mentioned that most published data reports that seroconversion was achieved by Day 14 after the 3rd dose of vaccine using PVRV under the ESSEN regimen [21, 22]. The effectiveness of this type of PEP in healthy patients and in pregnant women was discussed [21, 22]. Unfortunately, follow-up with a significant number of volunteers, especially those with certain medical conditions, could not be continued for more than 35 days after vaccination. Based on the results, the current study conclusively determined that the traditional ESSEN protocol using PVRV vaccine is sufficient for rabies prophylaxis in these patients. However, further studies on more patients with each medical condition considered in this study will provide more useful data.
