# Correction: Neutralizing Antibody Response after Intramuscular Purified Vero Cell Rabies Vaccination (PVRV) in Iranian Patients with Specific Medical Conditions

**DOI:** 10.1371/journal.pone.0142244

**Published:** 2015-10-30

**Authors:** Pooneh Rahimi, RouhAllah Vahabpour, Mohammad Reza Aghasadeghi, Syed Mehdi Sadat, Nader Howaizi, Ehsan Mostafavi, Ali Eslamifar, Vida Fallahian

There are errors in the author affiliations. The affiliations should appear as shown here:

Pooneh Rahimi^1^, RouhAllah Vahabpour^1^, Mohammad Reza Aghasadeghi^1^, Syed Mehdi Sadat^1^, Nader Howaizi^2^, Ehsan Mostafavi^4^, Ali Eslamifar^3^, Vida Fallahian^3^

**1** Department of Hepatitis and AIDS, Pasteur Institute of Iran, Tehran, Iran, **2** WHO Collaborating Center for Reference and Research on Rabies, Pasteur Institute of Iran, Tehran, Iran, **3** Department of Vaccination, Rabies post-exposure prophylaxis, Pasteur Institute of Iran, Tehran, Iran, **4** Department of Epidemiology, Pasteur Institute of Iran, Tehran, Iran.

## References

[1] Rahimi P, Vahabpour R, Aghasadeghi MR, Sadat SM, Howaizi N, Mostafavi E, et al (2015) Neutralizing Antibody Response after Intramuscular Purified Vero Cell Rabies Vaccination (PVRV) in Iranian Patients with Specific Medical Conditions. PLoS ONE 10(10): e0139171 doi: 10.1371/journal.pone.0139171 26440665 10.1371/journal.pone.0139171 PMC4595008

